# Outcome and Toxicity of an Ifosfamide-Based Soft Tissue Sarcoma Treatment Protocol in Children. The Importance of Local Therapy

**DOI:** 10.1080/13577149877939

**Published:** 1998-12

**Authors:** S. Murray Yule, Roderick Skinner, Martin W. English, Mike Cole, Andrew D. J. Pearson, Helen H. Lucraft, Alan W. Craft

**Affiliations:** 1 Department of Child Health The University of Newcastle upon Tyne UK; 2 Department of Pharmacological Sciences The University of Newcastle upon Tyne UK; 3 Department of Statistics The University of Newcastle upon Tyne UK; 4 The Northern Centre for Cancer Treatment Newcastle General Hospital Newcastle upon Tyne UK; 5 Department of Haematology Yorkhill NHS Trust Glasgow G3 8SJ UK

## Abstract

*Background.* Although the survival of children with soft tissue sarcoma (STS) has improved considerably, the outcome of patients with metastatic disease, and those with primary tumours of the extremities or parameningeal sites remains disappointing. We describe the clinical outcome of an ifosfamide-based regimen with local therapy directed only to children who failed to achieve a complete response to initial chemotherapy.

*Patients and Methods.* Twenty-one children with STS (16 rhabdomyosarcoma) who presented with unresectable tumours were treated with five courses of ifosfamide (9 g/m^2^) and etoposide (600 mgm^2^). Patients who did not achieve a complete response then received local therapy. Chemotherapy with ifosfamide combined with etoposide, vincristine (1.5 mg/m^2^ and doxorubicin (60 mg/m^2^) or vincristine and actinomycin D (1.5 mg/m^2^) was continued for one year.

*Results and Discussion.* Objective responses to five courses of ifosfamide and etoposide were seen in all patients. Disease free survival (DFS) at a median follow up of 59 months was 57% (95% CI 29–75%). The DFS of children who received local therapy was 89% compared with 33% in those who received chemotherapy alone (*p*=0.027). Locoregional recurrences did not occur in children who received radiotherapy to the site of the primary tumour. Ifosfamide-based chemotherapy does not reduce the incidence of loco-regional recurrence in children who do not receive local therapy.


*Sarcoma* (1998) **2**, 171–177

ORIGINAL ARTICLE

### Outcome and toxicity of an ifosfamide-based soft tissue sarcoma treatment protocol in children. The importance of local therapy

S. MURRAY YULE,[1,2] RODERICK SKINNER,[1] MARTIN W. ENGLISH,[1] MIKE COLE,[1,3] ANDREW D. J. PEARSON,[1] HELEN H. LUCRAFT[4] & ALAN W. CRAFT[1]

[1]*Departments of Child Health,* [2]*Pharmacological Sciences &* [3]*Statistics, The University of Newcastle upon Tyne &* [4]*The Northern Centre for Cancer Treatment, Newcastle General Hospital, Newcastle upon Tyne*

**Abstract**

*Background.* Although the survival of children with soft tissue sarcoma (STS) has improved considerably, the outcome of patients with metastatic disease, and those with primary tumours of the extremities or parameningeal sites remains disappointing. We describe the clinical outcome of an ifosfamide-based regimen with local therapy directed only to children who failed to achieve a complete response to initial chemotherapy.

*Patients and Methods* Twenty-one children with STS (16 rhabdomyosarcoma) who presented with unresectable tumours were treated with five courses of ifosfamide (9 g/m$^2$) and etoposide (600 mg/m$^2$). Patients who did not achieve a complete response then received local therapy. Chemotherapy with ifosfamide combined with etoposide, vincristine (1.5 mg/m$^2$ and doxorubicin (60 mg/m$^2$) or vincristine and actinomycin D (1.5 mg/m$^2$) was continued for one year.

*Results and Discussion.* Objective responses to five courses of ifosfamide and etoposide were seen in all patients. Disease free survival (DFS) at a median follow up of 59 months was 57% (95% CI 29–75%). The DFS of children who received local therapy was 89% compared with 33% in those who received chemotherapy alone ($p = 0.027$). Locoregional recurrences did not occur in children who received radiotherapy to the site of the primary tumour. Ifosfamide-based chemotherapy does not reduce the incidence of loco-regional recurrence in children who do not receive local therapy.

**Key words:** *soft tissue sarcoma, chemotherapy, local control.*

### Introduction

Soft tissue sarcomas (STS) constitute up to 6% of all childhood malignancies. The overall survival for children with rhabdomyosarcoma (RMS), the most common STS, has increased considerably over the last twenty-five years to 70%.[1] This improvement follows the introduction of multiagent chemotherapy combined with effective treatment of residual tumour masses with either surgery, radiotherapy (RT), or a combination of both.[2,3] The outcome of several subgroups of patients with RMS remains disappointing. Features consistent with a poor prognosis include the presence of metastatic disease,[3] high proliferative activity[4] and the presence of the [t(2;13)(q35;q14)] translocation.[5] Alveolar histology, previously thought of as being associated with treatment resistance, may be of lesser significance following the intensification of chemotherapy regimens.[3] The primary site of disease is also important with paratesticular and orbital tumours having a more favourable prognosis than those located in the parameningeal regions of the head and neck or the extremities.[2,3]

The rarity of non-RMS STS has led to uncertainty over the optimal treatment for these tumours. Although generally considered to be less chemosensitive than RMS, many are treated in a similar manner with greater emphasis being placed on local disease control.[6] Two STS staging systems have been widely reported in the literature. The post-operative clinical grouping strategy of the Intergroup Rhabdomyosarcoma Study Group (IRS) has been criticised as being strongly influenced by the surgical approach employed in each case. An alternative approach has been adopted by the International Society of Paediatric Oncology (SIOP), which allocates treatment according to stage and post-surgical status. One consequence of the improved survival of children with STS has been an increasing awareness of the long term sequelae of treatment. These include the adverse effects of RT on growing bones, teeth, eyes, bladder and the pituitary gland, particularly in very young children.[7–11] The introduction of

Correspondence to: Dr S. M. Yule, Department of Haematology, Yorkhill NHS Trust, Glasgow G3 8SJ, UK. Tel: 0141 201 0000; Fax: 0141 201 0857.

1357-714X/98/030171-07 $9.00 © 1998 Carfax Publishing Ltd

**Table 1.** *Patient details*

| Patient | Age (years) | Tumour | Site | IRS | TNM |
|---|---|---|---|---|---|
| 1 | 0.6 | RMS(e) | infratemporal fossa* | III | $T_{2b}N_0M_0$ |
| 2 | 2 | RMS(e | middle ear* | III | $T_{2b}N_0M_0$ |
| 3 | 2 | RMS(l) | paratesticular | II | $T_{1b}N_0M_0$ |
| 4 | 2 | RMS(e) | scalp | III | $T_{1b}N_1M_0$ |
| 5 | 3 | RMS(e) | chest wall | III | $T_{2b}N_0M_0$ |
| 6 | 3 | RMS(e) | middle ear* | III | $T_{1b}N_0M_0$ |
| 7 | 3 | RMS(e) | intratemporal fossa* | III | $T_{2b}N_1M_0$ |
| 8 | 4 | RMS(sa) | chest wall | III | $T_{2b}N_1M_0$ |
| 9 | 4 | RMS(e) | genitourinary | III | $T_{2b}N_0M_0$ |
| 10 | 4 | RMS(e) | middle ear* | III | $T_{2b}N_0M_0$ |
| 11 | 5 | RMS(e) | orbit | III | $T_{1a}N_0M_0$ |
| 12 | 6 | RMS(e) | orbit | III | $T_{1ab}N_0M_0$ |
| 13 | 6 | RMS(e) | retroperitoneal | III | $T_{2b}N_0M_0$ |
| 14 | 7 | RMS(l) | soft palate | III | $T_{1ab}N_0M_0$ |
| 15 | 8 | RMS(a) | forearm | III | $T_{1b}N_1M_1$ |
| 16 | 14 | RMS(e) | genitourinary | III | $T_{2b}N_0M_0$ |
| 17 | 0.6 | peripheral nerve sheath tumour | pelvis | III | $T_{2b}N_0M_0$ |
| 18 | 2 | epithelioid schwannoma | face | III | $T_{1a}N_1M_0$ |
| 19 | 5 | angiosarcoma | pelvis | III | $T_{2b}N_1M_0$ |
| 20 | 6 | granular cell tumour (myoblastoma) | pelvis | III | $T_{2b}N_1M_0$ |
| 21 | 12 | epitheliod sarcoma | hand | II | $T_{1a}N_1M_0$ |

IRS: Intergroup Rhabdomyosarcoma Study clinical grouping.
TNM: Tumour Nodes Metastases staging system.
RMS: Rhabdomyosarcoma.
(e): embryonal.
(l): ileomyomatous.
(a): alveolar.
(sk): solid alveolar.
*parameningeal site.

more intensive chemotherapy has been accompanied by the appearance of second malignancies and may lead to a higher incidence of infertility.[12,13]

Despite improved survival the optimum combination of chemotherapy and timing of local disease control remains uncertain. Here we report the results of a ifosfamide-based treatment regimen in children who presented with tumours which were not completely resectable at presentation without mutilating surgery.

### Patient details

Twenty-one children (8 female) with STS (16 RMS) were treated between November 1987 and January 1994 using the treatment protocol described below (Table 1). The median age at diagnosis was 4 years (range 0.6–14 years). Eighteen children (85%) presented with unresectable disease, either as a consequence of the site of the primary tumour or as the result of locoregional or distant spread. Three patients (14%) had macroscopic/microscopic evidence of tumour following primary surgery (Nos. 3, 14 and 21). A single patient had widespread bone and bone marrow metastases at diagnosis (No. 15). No other patient had evidence of disseminated disease at presentation. Five children with RMS had primary tumours located at parameningeal sites, one of whom had evidence of direct intracranial extension on computed tomographic Imaging (No. 10). None of these patients had malignant cells present in a cytospin preparation of their cerebrospinal fluid. Two RMS tumours exhibited alveolar histology and the translocation [t(2;13)(q35;q14)] was identified in metaphase preparations from both (Nos. 8 and 15). An identical translocation was also detected in an epithelioid schwannoma (No. 18). No patient had received previous chemotherapy or RT.

### Treatment protocol

All children initially received five courses of Ifosfamide (3 g/m$^2$) combined with etoposide (200 mg/m$^2$) on each of three consecutive days (IE). This combination was administered for five courses prior

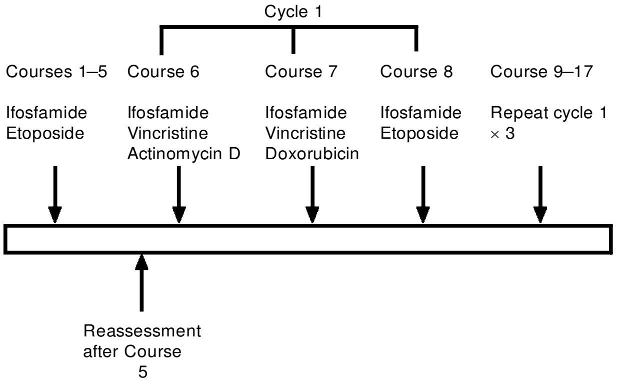

**Fig. 1.** *Outline of treatment regimen.*

to reassessment of the tumour. During these and subsequent courses Ifosfamide was administered as a continuous intravenous infusion over 72 hours accompanied by an equivalent dose of mesna included in the hydration fluid which was given at 3 $l/m^2$/day. Mesna hydration was continued for 12 hours after the infusion of ifosfamide was complete. Following five courses of IE further chemotherapy consisted of rotating courses of Ifosfamide (9 $g/m^2$) with vincristine (1.5 mg/m.3) and actinomycin D (1.5 $mg/m^2$ (IVA), ifosfamide with vincristine (1.5 $mg/m^2$) and doxorubicin (20 $mg/m^2$) administered over 6 hours on each of three consecutive days (IVAd) and further courses of IE (Fig. 1). Chemotherapy was administered at three weekly intervals or delayed until the neutrophil count had recovered to $1 \times 10^9$/l and the platelet count was greater than $100 \times 10^9$/l. Total treatment duration was one year. Chemotherapy was continued during RT but courses of IVA or IVAd were replaced with IE, and the order of subsequent courses was rearranged to ensure that each child received the same cumulative dose of each drug. In total, 21 children received a median of 15 courses of chemotherapy (range 9–18 courses) comprising a median of 126 $g/m^2$ of ifosfamide (range 81–162 $g/m^2$) and a median of 240 $mg/m^2$ of doxorubicin (range 0–240 $mg/m^2$. Children with parameningeal RMS received eight doses of intrathecal chemotherapy comprising cytosine arabinoside (30 mg), hydrocortisone (30 mg) and methotrexate (12.5 mg). In all cases response was evaluated by computerised axial tomographic scans with enhancement.

Responses were defined as follows: Complete Response (CR) resolution of all apparent tumour, Partial Response (PR) more than 50% decrease in tumour dimensions as measured by the sum of the products of the maximal perpendicular diameters of all measurable lesions and Stable Disease less than 50% decrease in tumour dimensions or no change.

### *Protocol violations*

Both children with orbital RMS achieved a CR with five courses of IE (Nos. 11 and 12). In view of this response, and the favourable prognosis of this tumour site a further four courses of IE only were administered. Another patient underwent tumour resection after 10 courses of chemotherapy. No viable tumour was found and therapy was discontinued in accordance with parental wishes (No. 17).

### *Local therapy*

After completing five courses of IE, 13 children (62%) (nine RMS) had residual macroscopic disease (Table 2). Six of these (four RMS) received RT at a median dose of 45 Grays (range 43–50 Grays) to the tumour bed with margins of at least 2 cm. The patient with an intracranial extension of her parameningeal RMS received an additional 30 Grays whole brain RT. Six children underwent surgical resection of residual tumour.

### Results

### Response

Re-evaluation following five courses of chemotherapy demonstrated that all patients had objective responses to IE (see Table 2). There were five CRs (four RMS) (24%) and 13 PRs (10 RMS) (62%). The Kaplan-Meier estimate of disease free survival (DFS) at a median follow up of 59 months (range 35–115 months) was 57% (95% CI 29–75%).[16] DFS in children who received appropriate local therapy, either surgery or RT, was 89% at 3 years compared to only 33% in those who received chemotherapy alone ($p = 0.027$, Log-rank test). Two children who developed recurrent disease without previously having received definitive local therapy were treated with further chemotherapy and RT. One is disease-free 17 months from recurrence (No 14). The second patient developed a further local recurrence 6 months later and is presently receiving palliative chemotherapy only (No 4).

### *Disease progression*

In all there were five locoregional recurrences (24%). Four of these children were receiving treatment for RMS. A single patient developed pulmonary metastases after completing treatment. The patient with multiple bony metastases from a RMS improved during therapy but deteriorated upon its completion. Of the five children who achieved a CR with chemotherapy none received definitive local therapy and two have subsequently developed disease recurrence at the site of the primary tumour.

One patient who was receiving treatment for a chest wall RMS accompanied by extensive mediastinal and para-aortic lymphadenopathy achieved a

**Table 2.** *Treatment outcome*

| Patient | Response to five courses of IE | Local therapy | Outcome | GFR | NTX |
|---|---|---|---|---|---|
| 1 | PR | none | locoregional recurrence | NP | NP |
| 2 | PR | RT | septic death | NP | NP |
| 3 | NE | surgery | disease free | 76 | 9 |
| 4 | CR | none | locoregional recurrence | NP | NP |
| 5 | PR | surgery | pulmonary metastases | NP | NP |
| 6 | PR | RT | disease free | 81 | 6 |
| 7 | PR | RT | disease free | 81 | 5 |
| 8 | PR | RT* | locoregional recurrence | NP | NP |
| 9 | PR | surgery | disease free | 98 | 1 |
| 10 | PR | RT | disease free | 76 | 3 |
| 11 | CR | none | disease free | 81 | 7 |
| 12 | CR | none | disease free | 75 | 2 |
| 13 | CR | none | disease free | 67 | 7 |
| 14 | NE | surgery | locoregional recurrence | 68 | 5 |
| 15 | PR | none | progressive disease | NP | NP |
| 16 | PR | none | septic death | NP | NP |
| 17 | PR | surgery | disease free | 74 | 5 |
| 18 | CR | none | locoregional recurrence | 87 | 10 |
| 19 | PR | surgery | disease free | 100 | 4 |
| 20 | PR | RT | disease free | 68 | 8 |
| 21 | NE | surgery | disease free | 59 | 8 |

Patients evaluated after five courses of ifosfamide/etoposide (IE). Follow up measurements of renal function performed 2 years following diagnosis.
RT: radiotherapy.
NP: not performed.
NE: not evaluable.
GFR: Glomerular filtration rate (ml/min/1.72 $m_2$).
NTX: nephrotoxicity score (Skinner *et al.* 1993).
0 = no nephrotoxicity.
1–3 = mild nephrotoxicity.
4–7 = moderate nephrotoxicity.
$\geq 8$ = severe nephrotoxicity.
*radiotherapy to affected lymph nodes only.

complete disappearance of his primary tumour and a considerable reduction in the size of his lymphadenopathy with five courses of IE (No. 8). In the absence of persisting disease at the primary site and in order to limit cardiac exposure, RT was administered to the site of his involved lymph nodes only. He developed a recurrence at the site of his primary tumour two months after completing chemotherapy. Another patient who presented with a parameningeal RMS at the age of 1 year achieved a PR with five courses of IE (No. 1). The likelihood of severe long-term post-RT sequelae in this child was considered to be high. A repeat biopsy found no evidence of viable tumour and no local therapy was administered. This child developed a locoregional recurrence five months after completing chemotherapy. Locoregional recurrences did not occur in children who received RT to the site of the primary tumour.

### Toxicity

### *Haematological and infective*

A total of 293 courses of chemotherapy were administered, 171 (58%) of these were complicated by an admission for fever in the presence of neutropenia. There were 41 independent septicaemic episodes with a variety of infecting bacteria. Twenty (49%) of these proven infections were related to indwelling central venous catheter colonization. Two children developed episodes of microbiologically proven fungal septicaemia. There were two deaths from overwhelming sepsis. Common toxicity criteria grade 3 thrombocytopenia and grade 4 neutropenla were invariable following chemotherapy. Haematological and infective toxicity was unaffected by concurrent RT but was more severe during the second 6 months of treatment. All children required intermittent support with blood products.

*Gastrointestinal*

Chemotherapy induced nausea and vomiting were manageable with ondansetron combined with dexamethasone. Weight loss during therapy was invariable and seven children (33%) required nasogastric alimentation. Constipation and anal fissures were common.

*Renal*

Fifteen children were available for renal function testing 2 years from diagnosis. The median glomerular filtration rate was 76 mils/min/1.73m$^2$ (range 59–100 mls/min/1.73m$^2$) (see Table 2). A combined score of both glomerular and tubular function in these children produced a median value of 5 (range 1–10), with 4 to 7 representing moderate nephrotoxicity and greater than or equal to 8 severe nephrotoxicity.[14] Detailed description of the extent of both glomerular and tubular dysfunction In many of these patients has been presented elsewhere.[15]

### Other toxicities

Whilst increased somnolence during IF therapy was common, this rarely extended beyond 50% of waking hours (WHO Grade 2). Severe somnolence and confusion followed three courses of chemotherapy in three different patients (WHO Grade 3). In each case neurological impairment resolved spontaneously and did not recur. Grade 3 neurotoxicity was not considered an indication for adjusting subsequent chemotherapy. Local reactions to RT were uncommon and no more severe than expected despite continuing chemotherapy with IE. Cardiac toxicity was monitored using echocardiography and no patient has a persistent fractional shortening of less than 30%. Haemorrhagic cystitis requiring admission and platelet support occurred in two children. The patient who received cranial irradiation has persisting alopecia. Following chemotherapy one patient developed a symptomatic oesophageal stricture as a consequence of severe mucositis, and continues to require regular dilatation. Two children who received RT for a RMS of the middle ear have marked unilateral hearing loss. There have been no second malignancies.

### Discussion

STS of childhood represent a diverse group of tumours which differ widely in terms of their malignant potential and treatment response. RMS, the largest single member of this group, is increasingly recognised to be a spectrum of different diseases rather than a single entity. This is reflected in the variable prognosis for RMS at different anatomical sites.[2,3] The location of the primary tumour does not provide a complete explanation as to why one child fares well with existing therapy, whilst another patient with a histological and anatomically identical tumour develops recurrent disease. In the future this issue may be clarified by molecular studies.[17] The presence of a chromosomal translocation [t(2;13)(q35;q14)] in RMS has been linked with the presence of disseminated disease at diagnosis and a poor response to treatment.[18] This translocation was detected in three patients, all of whom died from recurrence.

Here we report the use of an ifosfamide-based treatment protocol in the management of all non-resectable sarcomas. At its inception this programme was designed to improve cure rates In children who presented with tumours which were unresectable without mutilating surgery. Five courses of IE were given to shrink the tumour mass prior to definitive local therapy. Chemotherapy was continued for 1 year in an effort to eradicate systemic micrometastases. The conclusions that can be drawn from these results are limited by both the small number of patients and the heterogeneity of the tumours treated. The Introduction of Ifosfamide as the alkylating agent ‘backbone’ of this regimen followed several promising reports of its efficacy In the treatment of STS and possible superiority over cyclophosphamide.[19–26] An earlier report demonstrated the safety of 9 g/m$^2$ of ifosfamide when administered over three days in children with normal renal function.[27] Etoposide and ifosfamide are synergistic *in vitro* and the combination has excellent activity against STS in phase II studies.[28] This activity was evident in the 100% response rate to five courses of IE. The sensitivity of STS to vincristine, actinomycin D and doxorubicin is well established.[29–31]

Actinomycin D and doxorubicin are known radiosensitisers and the administration of either of these drugs during RT is accompanied by an increase in radiation-induced local tissue damage.[32] This has led to both drugs being avoided during and immediately following RT. Although several studies have shown that etoposide inhibits the repair of sub-lethal radiation damage in experimental systems there is little evidence of an adverse reaction between epodophyllotoxins and RT in clinical practice.[33,34] As a consequence of this we chose to continue IE during RT rather than reduce the intensity of chemotherapy delivered. This approach was not accompanied by an increase in the severity of local radiation toxicity. Although this finding requires confirmation in a larger study we suggest that the combination of IE and RT, at the doses employed here, does not lead to prohibitive radiation toxicity.

Our treatment regimen produced an overall DFS of 57% at a median follow up of 59 months. The DFS of children who received definitive local therapy in this study was superior at 89%. This suggests that overall treatment efficacy may have been compromised by the omission of local therapy from children who exhibited an excellent initial response to chemotherapy in an effort to reduce long-term sequelae. The traditional view of definitive local therapy being necessary to eradicate STS is being challenged in an effort to reduce the long-term sequelae of treatment. Current SIOP studies reserve surgery and RT for children with parameningeal RMS and patients who fail to achieve a CR with chemotherapy alone. Two of the five children in this study who did not receive local therapy developed recurrent disease at the site of the primary tumour. Only three children, two with orbital RMS, a particularly favourable prognostic group, and one patient with retroperitoneal RMS remain disease free after chemotherapy alone.

The results presented here indicate that, despite intensifying the chemotherapy of STS, local therapy remains important in the prevention of locoregional recurrence. No patient in this study who received definitive surgery or RT to the site of the primary tumour developed a local recurrence. These results highlight the dangers of withholding local therapy on the basis of imaging or repeat biopsy studies following an apparent CR to primary chemotherapy, except possibly in the case of orbital RMS. The decision to avoid RT in children with disease at other sites, especially elsewhere in the head and neck, following a CR to chemotherapy should be taken with caution and should be explored in a larger multicentre study. It has been suggested that decisions regarding further therapy are based upon the initial response to chemotherapy.[26] We would advocate caution in this respect, particularly in making decisions about the necessity for surgery or RT. Two out of five children in this study who achieved a CR with chemotherapy, in the absence of local treatment subsequently developed a locoregional recurrence and died.

The toxicity of this regimen was considerable. Two children died from overwhelming sepsis (9%), a proportion which is consistent with reports from other studies.[3,35] At the time of introduction of this treatment regimen ifosfamide-nephrotoxicity was unknown with early reports of ifosfamide chemotherapy in children either failing to recognise renal toxicity or underestimating its severity. Eight years later we recognise the importance of this problem.[14] Overall the high incidence of long-term nephrotoxicity reported here reflects the large cumulative dose of ifosfamide administered to many of the patients.[15] In contrast to the relatively high administered cumulative dose of ifosfamide, the total dose of doxorubicin was only 240 mg/m$^2$ thus explaining the absence of significant cardiotoxicity. A low incidence of significant neurotoxicity was seen and is consistent with previous reports of the rarity of this condition in children. Our approach of not altering subsequent ifosfamide chemotherapy following encephalopathy was justified by a zero recurrence rate.

This report demonstrates that despite intensive multiagent chemotherapy a high rate of loco-regional recurrence was seen amongst children with STS who did not receive definitive local therapy. This may have compromised overall survival. Patients who completed ifosfamide chemotherapy with appropriate local therapy did well but many have significant nephrotoxicity. The initial response to IE was impressive and warrants comparison with other regimens. We do not advocate the widespread introduction of this treatment protocol because of its considerable toxicity, in particular its nephrotoxicity. Instead, we believe this report illustrates the difficulty in deciding which patients require local therapy and highlights the dangers of withdrawing RT in favour of an Impressive response to a new chemotherapy regimen. Further progress in optimising treatment regimens for STS will follow the completion of ongoing multicenter studies.

### Acknowledgements

The authors would like to thank the North of England Childrens Cancer Research fund for their assistance. S. M. Yule was supported by the Tyneside Leukaemia Research Fund and R. Skinner was a Medical Research Council training fellow. M. W. English was supported by funding from ASTA Medica, Frankfurt and the Newcastle Health Authority. The authors are grateful to Dr M. C. G. Stevens for his comments on the manuscript.

### References

1 Crist WM, Kun LE. Common solid tumours of childhood. *N Eng J Med* 1991; 324:461–7.
2 Rodary C, Gehan K, Flamant F, Treuner J, Carli M, Auquier A *et al.* Prognostic factors in 951 non-metastatic rhabdomyosarcoma in children: a report from the international rhabdomyosarcoma workshop. *Med Pedtatr Oncol* 1991; 19:89–95.
3 Crist WM, Gehan KA, Ragab AH, Dickman PS, Donaldson SS, Fryer C *et al.*, The third intergroup rhabdomyosarcoma study. *J Clin Oncol* 1995; 13:610–30.
4 Niggli FK, Powell JE, Parkes SE, Ward K, Raafat F, Mann JR *et al.* DNA ploidy and proliferative activity (S-phase) in childhood soft-tissue sarcomas: their value as prognostic indicators. *Br J Can* 1994; 69:1106–10.
5 Douglass EC, Shapiro DN, Valentine M, Rowe ST, Carroll AJ, Raney RB, *et al.* Alveolar rhabdomyosarcoma with the t(2;13): cytogenetic findings and clinicopathologic correlations. *Med Pediatr Oncol* 1993; 21:83–87.
6 Horowitz ME, Pratt CB, Webber BL, Hustu HO, Etcubanas K, Miliauskas J, *et al.* Therapy for childhood soft-tissue sarcomas other than rhabdomyosarcoma: a review of 62 cases treated at a single institution. *J Clin Oncol* 1986; 4:559–64.
7 Heyn RM, Ragab AH, Raney RB Jnr, Ruymann F, Tefft M, Lawrence W Jnr. Late effects of therapy in orbital rhabdomyosarcoma in children: a report from the intergroup rhabdomyosarcoma study. *Cancer* 1986; 57:1738–43.
8 Fromm M, Littman P, Raney RB, Nelson L, Handlers S, Diamond G, *et al.* Late effects after treatment of twenty children with soft tissue sarcomas of the head and neck. *Cancer* 1986; 60:2570–5.
9 Kao GD, Willi SM, Goldweln J. The sequellae of chemo-radiation therapy for head and neck cancer in children: managing impaired growth, development and other side effects. *Med Pediatr Oncol* 1993; 21: 60–6.
10 Raney RB, Heyn R, Hays DM, Tefft M, Newton WA Jnr, *et al.* Sequelae of treatment in 109 patients followed for 5 to 15 years after diagnosis of sarcoma of the bladder and prostate. A report from the Intergroup Rhabdomyosarcoma Study Committee. *Cancer* 1993; 71:2387–94.
11 Kaste SC, Hopkins KP, Bowman LC. Dental abnormalities in long-term survivors of head and neck rhabdomyosarcoma. *Med Pediatr Oncol* 1995; 25:96–101.
12 Aubier F, Flamant F, Brauner R, Caillaud JM, Chaussain JM, Lemerle J. Male gonadal function after chemotherapy for solid tumours in childhood. *J Clin Oncol* 1989; 7:304–9.
13 Heyn RM, Haeberlen V, Newton WA, Ragab AH, Raney RB, Tefft M. Second malignant neoplasms in children treated for rhabdomyosarcoma. *J Clin Oncol* 1993; 11:262–70.
14 Skinner R, Sharkey IM, Pearson ADJ, Craft AW. Ifosfamide, mesna and nephrotoxicity in children. *J Clin Oncol* 1993; 11:173–90.
15 Skinner R, Pearson ADJ, English MW, Price L, Wyllie RA, Coulthard MG, *et al.* Risk factors for ifosfamide nephrotoxicity in children. *Lancet* 1996; 348:578–80.
16 Kaplan EL, Meier P. Nonparametric estimation from incomplete observations. *J Am Stat Assoc* 1958; 53:457–61.
17 Pappo AS, Shapiro DN, Crist WM, Maurer HM. Biology and therapy of paediatric rhabdomyosarcoma. *J Clin Oncol* 1995; 13:2123–39.
18 Kelly KM, Womer RB, Sorensen PHB, Xiong Q-B, Barr FG. Common and variant gene fusions predict distinct clinical phenotypes in rhbdomyosarcoma. *J Clin Oncol* 1997; 15:1831–36.
19 Pinkerton CR, Rogers H, James C, Bowman A, Barbor PR, Eden OB, *et al.* A phase II study of ifosfamide in children with recurrent solid tumours. *Can Chem Pharmacol* 1985; 15:258–62.
20 de Kraker J, Voute PA. The role of ifosfamide in paediatric soft tissue sarcomas. *Can Chem Pharmacol* 1986; 18:(Supplement) S23–4.
21 Bramwell VH, Mouridsen HT, Santoro A, Blackledge G, Somers R, Verwey J, *et al.* Cyclophosphamide versus ifosfamide. Final report of a randomised phase II trial in adult soft tissue sarcomas. *Eur J Can Clin Oncol* 1987; 23:311–21.
22 Pinkerton CR, Pritchard J. A phase II study of ifosfamide in paediatric solid tumours. *Can Chem Pharmacol* 1989; 24:(Supplement) S13–5.
23 Pratt CB, Douglass EC, Etcubanas EL, Goren MP, Green AA, Hayes FA. Ifosfamide in paediatric malignant soft tumours. *Can Chem Pharmacol* 1984; 24:(Supplement) S24–7.
24 Treuner J, Koscielniak K, Keim M. Comparison of the rates of response to ifosfamide and cyclophosphamide in primary unresected rhabdomyosarcomas. *Can Chem Pharmacol* 1989; 24:(Supplement) S48–50.
25 Pappo AS, Etcubanas E, Santana VM, Rao BN, Kun LE, Fontanesi J, *et al.* A phase II trial of ifosfamide in previously untreated children and adolescents with unresectable rhabdomyosarcoma. *Cancer* 1993; 71:2119–25.
26 Treuner J. Ifosfamide in the treatment of soft tissue sarcoma. Experience of the German cooperative soft tissue sarcoma studies. *Am J Pediatr Haem Oncol* 1993; 15:(Supplement A) S21–6.
27 Davies SM, Pearson ADJ, Craft AW. Toxicity of high-dose ifosfamide in children. *Can Chem Pharmacol* 1989; 24:(Supplement) S8–10.
28 Miser JS, Kinsella TJ, Triche TJ, Tskos M, Jarosinski P, Forquer R, *et al.* Ifosfamide with mesna uroprotection and etoposide; an effective regimen in the treatment of recurrent sarcomas and other tumours of children and young adults. *J Clin Oncol* 1987; 5:1191–8.
29 James DH, George P. Vincristine in children with malignant solid tumours. *J Pedtatr* 1964; 64:534–41.
30 Tan CTC, Dargeon HW, Burchenal JH. Effect of actinomycin D in childhood cancer. *Pediatr* 1969; 24:544–61.
31 Pratt CB, Shanks EC. Doxorubicin in the treatment of malignant solid tumours in children. *Am J Dis Child* 1974; 127:534–6.
32 Phillips TL, Fu KK. Acute and late effects of multimodal therapy on normal tissues. *Cancer* 1977; 40:(Supplement 1) 489–94.
33 Minehan KJ, Bonner JA. The interaction of etoposide with radiation: variation in cytotoxicity with the sequence of treatment. *Life Sci* 1993; 53:PL237–42.
34 Ng CE, Bussey AM, Raaphorst GP. Inhibition of potentially lethal and sublethal damage repair by camptothecin and etoposide in human melanoma cell lines. *Int J Rad Biol Phys* 1994; 66:49–57.
35 Ortega JA, Ragab AH, Gehan EA, Donaldson SS, Wiener K, Webber B, *et al.* A feasibility, toxicity and efficacy study of ifosfamide, actinomycin D and vincrisine for the treatment of childhood rhabdomyosarcoma. *Am J Pediatr Hem Oncol* 1993; 15:(Supplement A) S15–20.
